# Adenocarcinoma in ectopic prostatic tissue at the trigone of urinary bladder

**DOI:** 10.1002/iju5.12608

**Published:** 2023-07-25

**Authors:** Shunsuke Mori, Takahiro Maekawa, Yuma Kujime, Mai Akiyama, Makoto Matsushita, Mototaka Sato, Norihide Tei, Osamu Miyake

**Affiliations:** ^1^ Department of Urology Toyonaka Municipal Hospital Osaka Japan

**Keywords:** adenocarcinoma, bladder metastasis, differential diagnosis, ectopic prostatic tissue, urinary bladder

## Abstract

**Introduction:**

Ectopic prostatic tissue is prostatic tissue located distant from the prostate gland. Although its existence is not uncommon, the occurrence of adenocarcinoma in ectopic prostatic tissue is rare.

**Case presentation:**

A 68‐year‐old man was suspected to have a nodular‐type tumor in the bladder trigone and a tumor in the prostate based on magnetic resonance imaging and cystoscopy results. Transurethral tumor resection and transrectal prostate needle biopsy revealed the coexistence of ectopic prostatic adenocarcinoma in the bladder trigone and low‐risk orthotopic prostate cancer. Four years later, the tumor evolved to intermediate‐risk prostate cancer during active surveillance, and the patient underwent prostatectomy with resection of the bladder trigone. Pathology indicated no residual ectopic prostatic tissue or adenocarcinoma at the bladder trigone.

**Conclusion:**

Adenocarcinoma in ectopic prostatic tissue is very rare; however, when found, the possibility of concurrent cancer in the prostate gland should be considered.

**Abbreviations & Acronyms**
EPT: ectopic prostatic tissue
MRI: magnetic resonance imaging
PCa: prostate cancer
PSA: prostate‐specific antigen
TURBT: transurethral resection of bladder tumor

**Keynote message**

Adenocarcinomas arising within ectopic prostatic tissue differ from prostate cancer metastases in that benign prostate tissue is present. This condition is frequently accompanied by orthotopic prostate cancer, as reported here. If identified, screening for cancer in the prostate gland may be recommended.

## Introduction

EPT is tissue histologically proven to be prostate that is located away from the prostate gland. EPT is common in the urinary bladder and urethra.^1^ However, adenocarcinomas in EPTs are very rare, with only a few reports.^2^, ^3^, ^4^, ^5^, ^6^, ^7^ Herein, we report a case of coexistence of adenocarcinoma in an EPT in the urinary bladder and the prostate gland.

## Case presentation

**Fig. 1:**
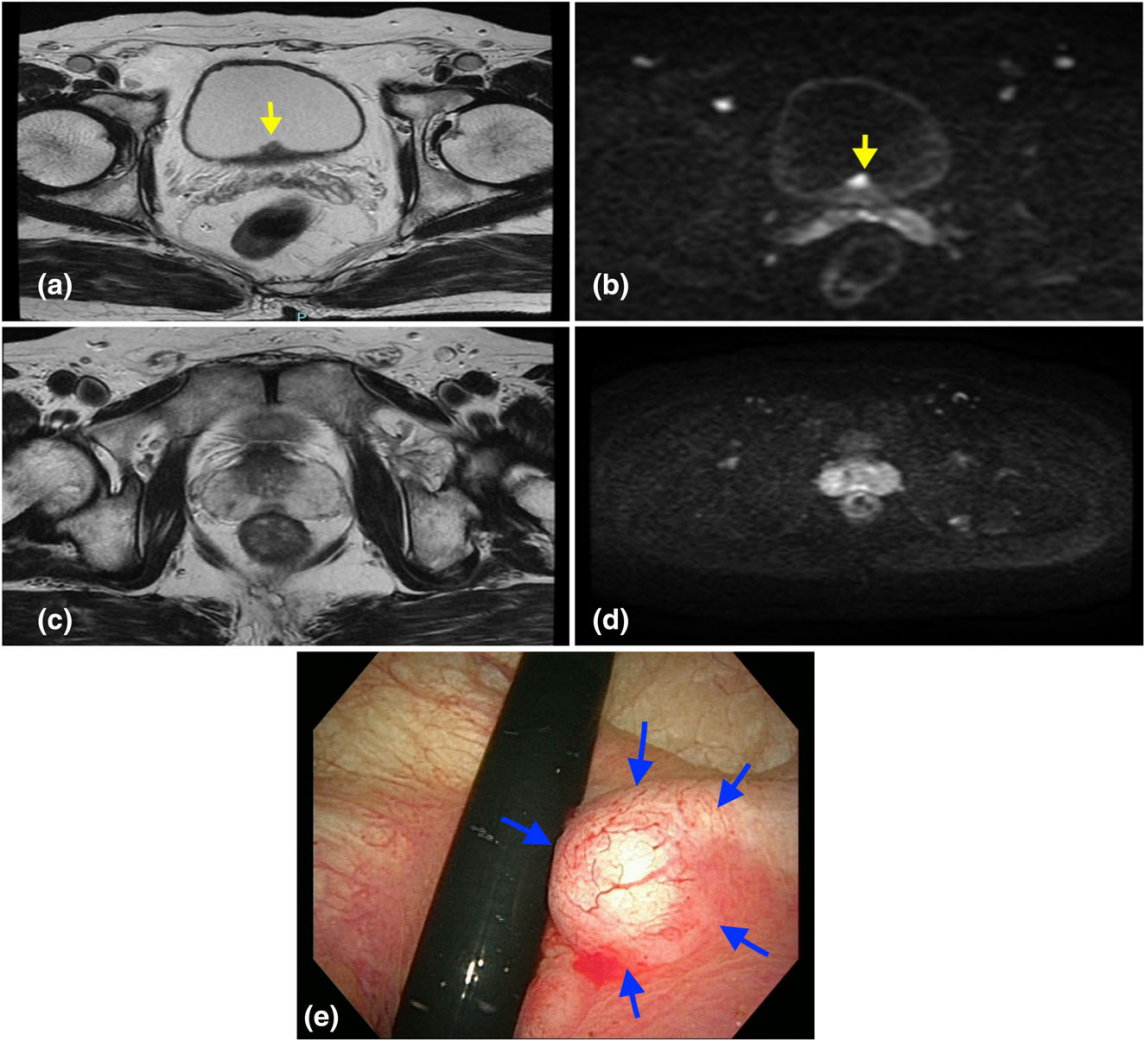
(a–e) Abdominal MRI and Cystoscopy image. (a) T2‐weighted imaging and (b) diffusion‐weighted imaging of the bladder. The yellow arrow indicates a nodular‐type tumor in the bladder trigone. (c) T2‐weighted imaging and (d) diffusion‐weighted imaging of the prostate, which show an area suspected to be PCa in the peripheral zone of right lobe of prostate. (e) Cystoscopy image showing a submucosal tumor in the bladder trigone (blue arrows).

**Fig. 2:**
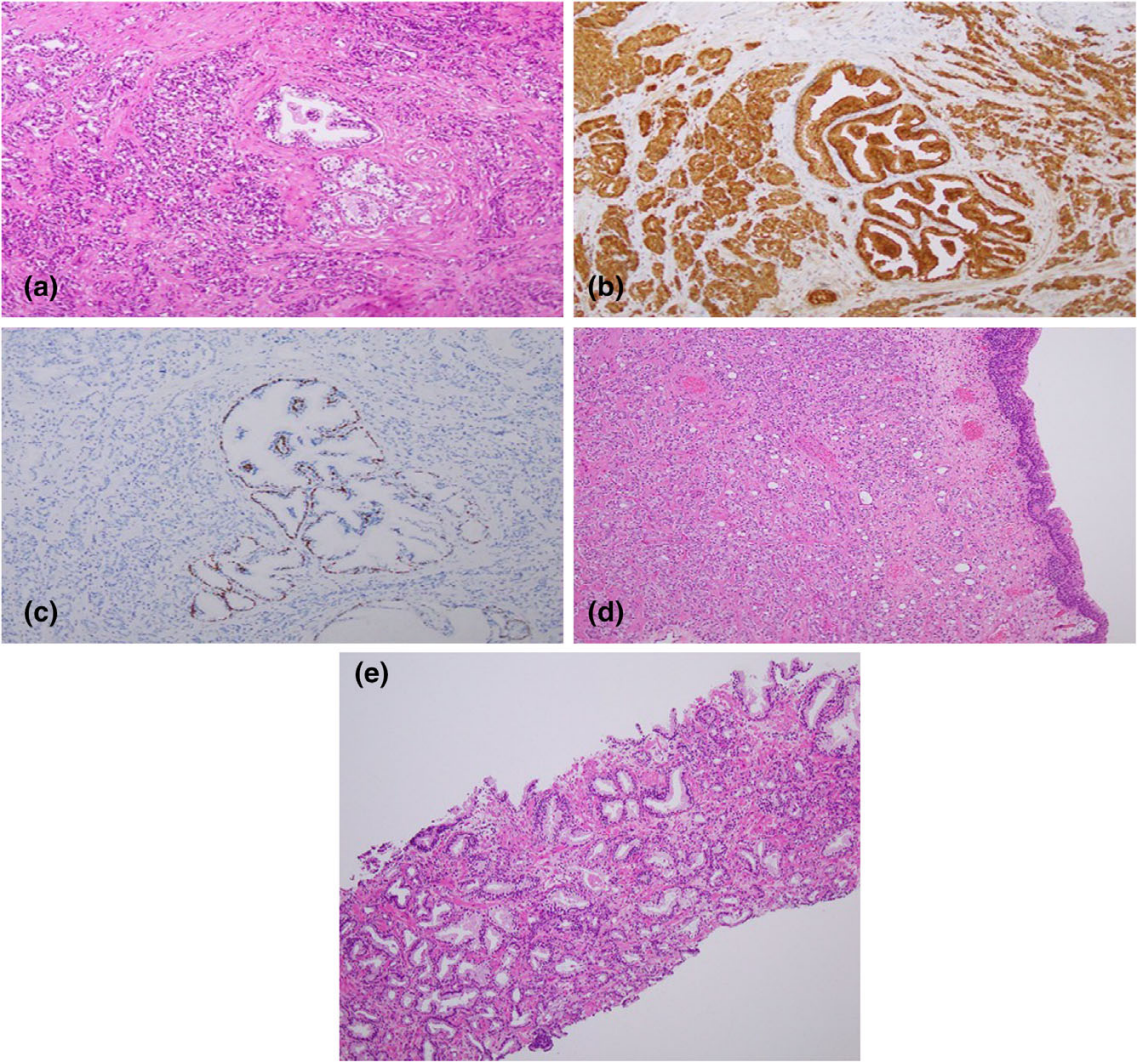
(a–e) Histopathological and immunohistochemical findings. Histopathology of the tumor in the bladder trigone revealed benign prostatic tissue and prostatic adenocarcinoma with a dominant Gleason Grade 4 pattern. (a) Hematoxylin and eosin staining, 40× magnification. (b) Positive immunostaining of PSA, 40× magnification. (c) Positive immunostaining of p40, which is a basal cell marker for benign prostatic glands, 40× magnification. (d) Histopathological findings indicating normal urothelial mucosa and adenocarcinoma‐infiltrated submucosa. Hematoxylin and eosin staining, 10× magnification. (e) Pathological examination of a transrectal prostate needle biopsy reveals prostate adenocarcinoma, Gleason Grade group 1, on hematoxylin and eosin staining, 10× magnification.

A 68‐year‐old man with no significant medical history was referred to our hospital because bladder and PCas were suspected based on abdominal MRI performed as part of a thorough medical checkup. MRI showed areas with low intensity on T2‐weighted imaging and high intensity on diffusion‐weighted imaging at the bladder trigone and in the peripheral zone of the right lobe of the prostate (Fig. 1a–d). His PSA values were within the normal range (2.839 ng/mL). Urinary cytology was negative for high‐grade urothelial carcinoma and cystoscopy revealed a submucosal nodular tumor in the bladder trigone (Fig. 1e). The patient underwent TURBT. Histopathological examination showed the existence of both benign prostatic tissue and a submucosal adenocarcinoma of Gleason Grade group 4 that was positive for PSA and negative for p50 (Fig. 2a–d) the tumor was not continuous with the prostate gland. Transrectal prostate needle biopsy was performed at the time of TURBT, and pathological examination indicated an adenocarcinoma of Gleason Grade group 1 in 3 of 12 specimens (Fig. 2e). Based on the overall findings, the patient was diagnosed with the coexistence of prostatic adenocarcinoma in the EPT in the urinary bladder and low‐risk orthotopic PCa (cT2aN0M0). Follow‐up TURBT 3 months later indicated no residual carcinoma in the urinary bladder. Active surveillance was chosen for low‐risk orthotopic PCa.

Four years after the diagnosis, a transrectal prostate needle re‐biopsy was performed because of elevated PSA (4.41 ng/mL). Pathological examination indicated that the tumor had progressed to Gleason Grade group 2, which upgraded the tumor to intermediate‐risk. The patient underwent robot‐assisted radical prostatectomy with resection of the scar in the bladder trigone. Pathological examinations revealed a diagnosis of pT2 acinar prostatic adenocarcinoma and no residual EPT or adenocarcinoma in the urinary bladder, which suggested that the TURBT performed 4 years previously was curative. The patient has maintained a low PSA level (<0.006 ng/mL) and has had no recurrence 3 months later.

## Discussion

EPT is prostate tissue located away from the prostate gland, confirmed by positive immunostaining for PSA, prostatic‐specific membrane antigen, or prostatic acid phosphatase, which are specific to prostatic tissue.^1^ One possible mechanism of EPT development is the embryologic migration of prostatic glands.^8^ Prostate tissue arises from the prostatic epithelium of the urethra in the fetus at approximately 10 weeks of gestation, invades the surrounding mesenchyme, and develops and differentiates to form the prostate gland.^9^ During the formation of the prostate gland, prostatic tissue may migrate into the submucosal area of the bladder trigone because such submucosa, which extends until the urethra, is of the same mesenchymal origin and embryologically continuous to the prostate. In our case, histopathological findings showed that the normal urothelial mucosa remained, and the adenocarcinoma had spread to the submucosa and muscularis of the bladder trigone. We suppose that EPT that had strayed into the submucosa of the bladder trigone developed adenocarcinoma, which appeared as a submucosal tumor. The occurrence of adenocarcinoma in EPTs is rare, with only 6 cases reported worldwide (Table 1. Most cases were asymptomatic, with one presenting hematuria, however, the patient presented concomitant bladder cancer, and whether the symptom was due to the ETP adenocarcinoma was unclear. Four of the cases were accompanied by prostate gland adenocarcinoma, as in this case.^2^, ^3^, ^4^, ^6^ One case revealed no malignancy in the prostate gland in a prostatic biopsy^5^; the last case had no prostate gland examination performed.^7^ That prostatic adenocarcinoma in EPT and orthotopic PCa tend to coexist is noteworthy. Several congenital and acquired factors may contribute to the risk of developing PCa.^10^, ^11^ In our case, regarding cogenetic predisposition, no family history of PCa was present, however, his father had cancer of unknown primary origin. According to the migration hypothesis, the EPT and orthotopic prostate originate from the same tissue, so we suppose that little differences exist between the EPT and orthotopic prostate malignancies regarding genetic characteristics. Additionally, acquired predispositions are the same. These considerations may signify that the risk for developing adenocarcinoma in the EPT and orthotopic prostate is identical. If a patient develops an adenocarcinoma in the orthotopic prostate, the possibility of developing an adenocarcinoma in the EPT exists. Therefore, upon diagnosis of an EPT adenocarcinoma, screening for malignancy in the prostate gland is recommended.

**Table 1:** Summary of the case reports of adenocarcinoma in EPTs

Author, year	Age	PSA (ng/mL)	Symptom	Ectopic PCa	Site of EPT	Orthotopic PCa
Adams *et al*.^2^ 1993	72	8.3	No	Grade group 1	Pararectal lesion	Grade group 1
Gardner *et al*.^3^ 2010	62	Not noted	Hematuria	Grade group 1	Bladder dome	Grade group 2
Backhouse *et al*.^4^ 2014	70	5.67	No	Grade group 2	Bladder dome	Grade group 2
Somwaru *et al*.^5^ 2016	80	13.1	No	Grade group 3	Seminal vesicle	No malignancy
Tolkach *et al*.^6^ 2017	68	2.48	No	Grade group 5	Retrovesical lesion	Grade group 2
Jeong *et al*.^7^ 2022	60	12.2	No	Grade group 1	Paravesical and pararectal lesion	Not noted
Our case 2023	68	2.839	No	Grade group 4	Bladder trigone	Grade group 1

When PCa is found in the bladder and is not contiguous with the prostate, as in this case, differentiating between PCa arising in EPT and bladder metastases of PCa is necessary. When EPT develops adenocarcinoma, benign prostatic tissue surrounding cancer should be present, which can differentiate EPT adenocarcinoma from bladder metastases of PCa. Such bladder metastases are rare, with only 9 cases reported (Table 2)^12^, ^13^, ^14^, ^15^, ^16^, ^17^, ^18^, ^19^, ^20^; the metastases were identified on MRI or computed tomography at the time of diagnosis of metastatic PCa or PSA elevation after radical prostatectomy or during hormonal therapy. In all cases, primary bladder cancer was initially suspected, yet TURBT revealed bladder metastases from PCa. If PCa is identified in the urinary bladder, the presence of benign prostatic tissue is key to differentiate EPT adenocarcinoma from bladder metastases.

**Table 2:** Summary of the case reports of bladder metastases from PCa

Author, year	Age	PSA at the time of bladder metastases (ng/mL)	PCa	Triggers for detecting bladder metastases	Metastatic sites
Buchholz *et al*.^12^ 1994	69	1.0	Grade group 2	Hematuria 14 months after radical prostatectomy with elevated PSA	Bladder
Uemura *et al*.^13^ 2001	67	3.4	Grade group 3	CT obtained for elevated PSA and hematuria 5 years after radical prostatectomy	Bladder, bone
Hallermeier *et al*.^14^ 2010	60	25.8	Grade group 3	CT obtained for elevated PSA during hormone therapy for PCa	Bladder, bone
Shirakawa *et al*.^15^ 2010	62	270.93	Grade group 3	MRI at diagnosis of PCa	Bladder, bone, lymph node
Kim *et al*.^16^ 2017	75	14.337	Not noted	MRI at diagnosis of PCa	Bladder
Imanaka *et al*.^17^ 2020	57	1.188	Not noted	acute urinary retention during hormone therapy for PCa	Bladder, lymph node
Okubo *et al*.^18^ 2020	74	0.23	Grade group 5	MRI obtained for elevated PSA and hematuria during hormone therapy for PCa	Bladder, lymph node
Spazzapan *et al*.^19^ 2022	79	29.9	Grade group 5	MRI at diagnosis of PCa	Bladder, bone, lymph node
Masuda *et al*.^20^ 2022	69	84	Grade group 5	CT obtained for elevated PSA during hormone therapy for PCa	Bladder, bone, lung, lymph node

## Conclusion

We reported a case in which ETP adenocarcinoma and orthotopic PCa coexisted. The existence of benign prostatic tissue around the adenocarcinoma in the urinary bladder guided the diagnosis of adenocarcinoma arising from EPT, instead of bladder metastases of PCa. Adenocarcinoma in EPTs is very rare; however, when present, the possibility of concurrent cancer in the prostate gland should be considered.

## Author contributions

Shunsuke Mori: Conceptualization; data curation; formal analysis; writing – original draft; writing – review and editing. Takahiro Maekawa: Data curation. Yuma Kujime: Data curation. Mai Akiyama: Data curation. Makoto Matsushita: Formal analysis; writing – review and editing. Mototaka Sato: Supervision. Norihide Tei: Supervision. Osamu Miyake: Supervision.

## Conflict of interest

The authors declare no conflict of interest.

## Approval of the research protocol by an Institutional Reviewer Board

Not applicable.

## Informed consent

Informed consent was obtained.

## Registry and the Registration No. of the study/trial

Not applicable.
